# Thyroid Hormone-Induced Angiogenesis

**DOI:** 10.2174/157340309787048158

**Published:** 2009-01

**Authors:** Paul J Davis, Faith B Davis, Shaker A Mousa

**Affiliations:** Ordway Research Institute, Inc., Albany, New York; † Pharmaceutical Research Institute, Albany College of Pharmacy, Albany, NY, USAOrdway Research Institute, Inc., Albany, New York

## Abstract

A series of reports in the past decade have ascribed pro-angiogenic activity to several thyroid hormone analogues, including L-thyroxine (T_4_), 3,5,3-triiodo-L-thyronine (T_3_) and diiodothyropropionic acid (DITPA). Model systems of angiogenesis have demonstrated that thyroid hormone-induced neovascularization is initiated at a cell surface receptor for the hormone on an integrin. The hormone signal is transduced within the cell by extracellular regulated kinase 1/2 (ERK1/2) into secretion of basic fibroblast growth factor (bFGF) and other vascular growth factors and consequent angiogenesis. Intact animal studies have shown that endogenous thyroid hormone supports blood vessel density in heart and brain and that thyroid hormone administration can induce angiogenesis in ischemic limbs.

## INTRODUCTION

In studies of thyroid hormone-induced myocardial hypertrophy a decade ago, Tomanek and co-workers showed that administration of L-thyroxine (T_4_) to rats caused left ventricular capillary growth [1]. This was accompanied by accumulation of basic fibroblast growth factor (*bFGF*) mRNA and bFGF protein. Because these changes occurred within several days of animal exposure to T_4_, when the hypertrophic response was small, the authors concluded in this study and in a follow-up report [1, 2] that the vascular and myocyte actions of thyroid hormone in this model represented separate effects of T_4_. The same laboratory subsequently found that an inotropic iodothyronine analogue, diiodothyropropionic acid (DITPA), increased abundance of vascular endothelial growth factor (VEGF) protein and angiopoietin-1 (Ang-1) in rat coronary arterioles, as well as bFGF [3]. There was no increase in ventricular mass in the treated animals. The molecular basis of the thyroid hormone effect on *bFGF* and other growth factor gene expression was not investigated. An effect of the thyroid hormone analogue on Ang-1 was puzzling, since this factor stabilizes quiescent endothelium, whereas Ang-2 is a de-stabilizing agent that prepares previously quiescent endothelium for the action of growth factors such as VEGF [4]. The structures of pro-angiogenic thyroid hormone analogues are shown in Fig. (**1**).

The cardiomyopathic hamster was recently shown by Kuzman *et al.* to respond to chronic DITPA administration with an increase in myocardial blood flow [5] consistent with increased angiogenesis. Subsequently, this group reported that *hypothyroidism* decreased blood vessel density in rat brain [6] and heart [7] and that administration of DITPA or T_4_ to thyroidectomized animals prevented blood vessel loss. Several clinical studies have asserted that thyroid hormone as L-thyroxine may be effective in heart failure [8, 9] but the state of the coronary circulation before and during treatment is not known. Clinical trials of DITPA in the management of heart failure, conducted by the Department of Veterans Affairs and Titan Pharmaceuticals, Inc., have been terminated (ClinicalTrials.gov), but a hypolipidemic trial continues.

The existence of a discrete effect of thyroid hormone analogues on angiogenesis has been documented in the chick chorioallantoic membrane model (CAM) [10, 11] and in the human dermal microvascular endothelial cell (HDMEC) microtubular model [11, 12]. The molecular basis of this effect has been defined and shown to be initiated at a cell surface receptor for thyroid hormone on endothelial cells that we have recently described [11, 13, 14]. Thus, a nuclear thyroid hormone receptor (TR) isoform [15] is not primarily involved in the pro-angiogenic action of the hormone.

The concept of thyroid hormone as a pro-angiogenic agent is relevant to the clinical use of hormone analogues as inotropic [16, 17] or cholesterol-lowering agents [18, 19]. Pro-angiogenic agents may also be of interest in accelerating wound-healing. Thyroid hormone has been reported to accelerate wound-healing, but this has been attributed to an action of the hormone on keratinocytes [20]. In the setting of cancer, in contrast, anti-angiogenesis is desirable and anti-angiogenic clinical strategies are being extensively explored [21, 22].

## THYROID HORMONE ACTION AT ITS CELL SURFACE RECEPTOR ON INTEGRIN αVβ3

In 2005, Bergh *et al.* demonstrated the existence of a high affinity thyroid hormone receptor on a structural plasma membrane protein, integrin αvβ3 [13]. A number of extracellular matrix (ECM) proteins are ligands of this heterodimeric protein [23] and the binding of each of these is a signal that is transduced by the integrin into an intracellular response (outside-in signaling). The protein can also transduce intracellular signals into extracellular events (inside-out signaling). The extracellular domain of this integrin includes an Asp-Gly-Asp (RGD) recognition site that is an important verification domain for the ligands of the protein; that is, each of the integrin ligands contain an RGD sequence that is required for the binding of each ECM protein ligand to its specific binding or receptor site on the integrin [23]. There may also be crosstalk between the RGD recognition site on αvβ3 and specific vascular growth factor receptors that may be clustered with the integrin [24, 25]. The initial description of the plasma membrane integrin receptor included evidence that the receptor mediated the effect of the hormone on angiogenesis [13], which had been previously reported [10].

The thyroid hormone receptor on integrin αvβ3 is located at the RGD recognition site and short RGD peptides interfere with the binding of thyroid hormone analogues to the integrin. T_4_ and 3,5,3’-triiodo-L-thyronine (T_3_) are agonist ligands at the hormone receptor site and are physiologically the most important thyroid hormone analogues. DITPA and GC-1 are iodothyronine analogues that also are agonist ligands at the integrin receptor [11, 14]. The thyroid hormone signal initiated at integrin αvβ3 is transduced by the mitogen-activated protein kinase (MAPK; extracellular regulated kinase, ERK1/2) cascade [10, 13]. The kinase activities upstream of MAPK for the thyroid hormone signal initiated at the cell surface have been identified [26], and several complex downstream events occur as a result of the activation of ERK1/2 by thyroid hormone. Such events may be local, e.g., regulation of the activity of the plasma membrane Na/H antiporter [27] that is important to myocardial function [28] or an increase in activity of cell membrane Na, K-ATPase (sodium pump) activity [29].

Other events initiated at the integrin involve several cellular compartments and include trafficking of proteins within the cell [30, 31] and complex cellular actions, such as angiogenesis [10]. Proteins in cytoplasm that translocate to the nucleus in response to the binding of thyroid hormone by the plasma membrane integrin receptor include activated ERK1/2 [26], and other signal transducing proteins [32, 33]. By this mechanism that does not primarily involve the nuclear receptor for thyroid hormone, the latter can amplify the intracellular responses to important systemic polypeptides that act at the plasma membrane. Examples of such polypeptides are interferon-γ [32] and epidermal growth factor-like ligands [33, 34].

The pro-angiogenic effect of thyroid hormone that is initiated at the integrin is induced by several thyroid hormone analogues, as noted above. The analogues are not interchangeable as angiogenic agents, however. For example, T_4_ acts on platelet integrin αvβ3 to cause agglutination [35], whereas T_3_, DITPA and GC-1 do not act on platelets. As a pro-angiogenic agent in the context of wound-healing, T_4_ would be more desirable than other analogues. In the setting of an ischemic organ or tissue, however, a pro- angiogenic agent should not promote aggregation of platelets. While T_3_ is more potent than T_4_ as an activator of cellular events that involve the intranuclear thyroid hormone receptor (TRβ1, TRα1), T_4_ and T_3_ are equipotent at the cell surface receptor and a case can be made at this receptor for the function of T_4_ as a hormone, rather than as a prohormone antecedent of T_3_ [36].

**Fig. (1):**
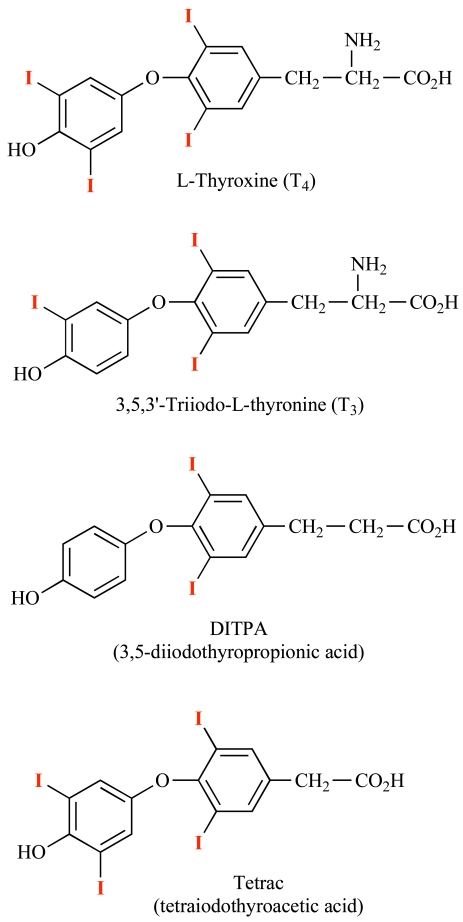
**Structures of thyroid hormone analogues**. L-Thyroxine (T_4_), 3, 5, 3’-triiodo-L-thyronine (T_3_) and DITPA (diiodothyropropionic acid) are pro-angiogenic and initiate angiogenesis at the integrin αvβ3 receptor for thyroid hormone. The receptor is located at the Arg-gly-Asp (RGD) recognition site on the integrin. Tetrac (tetraiodothyroacetic acid) is a deaminated derivative of T_4_ and is anti-angiogenic, blocking the binding of agonist thyroid hormone analogues at the integrin receptor. Tetrac is also capable of inhibiting the angiogenic actions of vascular endothelial growth factor (VEGF) and basic fibroblast growth factor (bFGF) in the absence of thyroid hormone. Action of the growth factors requires crosstalk between the integrin RGD recognition domain and the specific receptors for the VEGF and bFGF.

Tetraiodothyroacetic acid (tetrac) is a deaminated form of T_4_ (Fig. **1**) that is not an agonist at the receptor, but inhibits binding of agonist analogues to the integrin [13, 37]. Thus, tetrac is a useful probe of the participation of the receptor in actions of thyroid hormone analogues. Inside the cell, however, tetrac is a low-grade thyromimetic agent [38]. Thus, the most stringent application of tetrac as a marker of participation of the receptor in the actions of thyroid hormones is as re-formulations that cannot gain entry to the cell interior. One such re-formulation is nanoparticulate (poly[lactate-co-glycolic acid], PLGA) tetrac [39].

In addition to transduction of the iodothyronine signal vertically to the cell interior from the integrin receptor, the hormone-activated receptor on αvβ3 may alter the activities of specific polypeptide growth factor receptors that appear to be clustered with the integrin on the plasma membrane. For example, the epidermal growth factor (EGF)-like ligands bind to the cell surface EGF receptor (EGFR) and may have their signals amplified by action of thyroid hormone on the cell surface [34]. EGFR is found in heart cells [40] and EGFR signaling may be involved in myocardial hypertrophy [41]. In addition, EGFR signaling can result in VEGF and bFGF production [42], although this has been described in tumor cells and has not yet been looked for in myocardiocytes. These observations indicate the complexity of regulation of angiogenesis and imply that thyroid hormone can influence angiogenesis secondarily *via* the EGFR and an integrin receptor-EGFR interaction.

## INDUCTION OF ANGIOGENESIS BY THYROID HORMONE

Several model systems have been exploited to demonstrate the pro-angiogenic activity of thyroid hormone. As noted above, these initially have been the CAM and the HDMEC models and now include a rabbit hind limb ischemia model. Translation of studies involving cultured cells or the CAM assay into intact animal organ or tissue ischemia is a critical step in establishing the utility of the pro-angiogenic activity of thyroid hormone analogues. A rabbit hind-limb ischemia paradigm has been recently used in testing the angiogenic effect of thyroid hormone in an intact animal [43]. In these initial intact animal studies, increase in angiogenesis has been quantitated by measuring blood vessel buds on limb angiography and by the ratio of blood vessels to muscle fiber on muscle biopsy.

What are the steps that follow activation of MAPK (ERK1/2) by thyroid hormone at the cell membrane receptor and that lead to neovascularization? The sequence of events is incompletely understood, but is known to require importation of activated (tyrosine-phosphorylated) ERK1/2 into the nucleus and its consequence, transcription of the basic fibroblast growth factor (*bFGF*) gene. In the CAM assay, the addition of anti-bFGF protein blocks the pro-angiogenic effect of thyroid hormone [10]. We know that the vascular endothelial growth factor (*VEGF*) gene may also be transcribed in response to the binding of thyroid hormone by the integrin receptor [44]. Finally, accumulation of Ang-2, but not Ang-1, has been shown to occur in endothelial cells stimulated by thyroid hormone analogues *via* the cell surface receptor [45]. This is what is expected as a step preparatory to the action on endothelium of VEGF [4], in contrast to another report cited above in which the hormone enhanced tissue accumulation of endothelium stabilizing Ang-1 [3].

What transcription factor activities are modulated by activated MAPK is not yet known, but thyroid hormone-directed MAPK is known to activate (phosphorylate) members of the nuclear superfamily of hormone receptors, such as TRβ1 and estrogen receptor-α (ERα) [46, 47]. These superfamily members are transcription factors whose activation involves phosphorylation and liganding, respectively, of T_3_ or estradiol. T_3_ has been shown to increase expression of the *VEGF* gene in a model of liver regeneration [44, 48] and thyroid hormone administration to intact animals, as noted above, can increase *bFGF* expression [1].

If TR isoforms are in fact involved in the pro-angiogenic action of iodothyronines that begin at the cell surface, then this is an example of the interface of membrane-initiated actions and downstream effects involving TR in which thyroid hormone need not be present in the cell nucleus. *Nongenomic* is a term applied to hormone actions that do not primarily require intranuclear complexing of hormone and relevant superfamily nuclear receptor family member [37, 39]. Nongenomic actions may involve the iodothyronine receptor on plasma membrane integrin αvβ3, or nuclear TR isoforms that are now appreciated to reside in cytoplasm [39, 49].

The signal transduction and activator of transcription (STAT) family, such as STAT1α [32], and STAT3 [33], are also serine phosphorylated by thyroid hormone-activated ERK1/2. These cellular polyfunctional proteins are involved in the actions of a variety of cytokines [50], including the interferons, as noted above. While the STATs are activated primarily by tyrosine phosphorylation [51], specific serine phosphorylation of these proteins amplifies their transcriptional activity [26, 32]. The STAT proteins are involved in vascular growth factor biochemistry. For example, the VEGF signal is transduced by STAT1 [52] and STAT3 can be involved in VEGF gene expression [53] and either or both of these processes, based upon what we know about thyroid hormone, may be sites of hormone action that contribute to angiogenesis. The FGF receptor (FGFR) on the cell surface, when liganded, generates an intracellular signal that is STAT1-mediated [54]. Again, iodothyronines could amplify transduction of this signal and thus enhance angiogenesis. Finally, glycoprotein 130 (gp130) is an intracellular signaling protein activated in cardiac myocytes in the process of tissue hypertrophy. *Via* STAT3, gp130 can induce *VEGF* gene transcription [55] that is required for support of hypertrophy. This molecular signaling system is a candidate contributor to the studies mentioned above in which thyroid hormone induced myocardial hypertrophy [1] and one can speculate that amplification of STAT3 action by thyroid hormone-directed ERK1/2 might be the vehicle by which thyroid hormone can enhance angiogenesis whether or not cardiac hypertrophy is present. In summary, the STATs are attractive, but as yet unproved, candidate molecular mediators of thyroid hormone-induced neovascularization in the heart.

## REFORMULATED THYROID HORMONE ANALOGUES THAT ACT AT THE INTEGRIN RECEPTOR

Unmodified T_4_ and T_3_ can act at the cell surface or can be taken up by cells *via* transport systems [56, 57]. Within the cell or at the plasma membrane, T_4_ must be converted to T_3_ in order to have nuclear or mitochondrial effects [57-59]. However, T_4_ can affect the state of the cytoskeleton without conversion to T_3_; further, rT_3_, but not T_3_, can also effect conversion of soluble actin to fibrous (F) actin in the cell [60]. Metabolism of T_4_ or T_3_ to T_2_ may be important to regulation of oxidative phosphorylation in mitochondria [61].

Thyroid hormone must be reformulated in order to obtain analogues that act exclusively at the integrin. Thyroid hormone (T_4_ or T_3_) with an agarose tail, although not taken up by the cell, stimulates angiogenesis [10]. A thyroid hormone nanoparticulate analogue we have developed is T_4_-poly (lactate-co-glycolic acid) (PLGA), mentioned above, and this is ether-bonded to the outer ring hydroxyl of T_4_. This agent is pro-angiogenic in the CAM and HDMEC microtubular models (Mousa SA, unpublished observations). Thus, the outer ring hydroxyl is not critical to the angiogenic action of the hormone.

## POSSIBLE APPLICATIONS OF THYROID HORMONE ANALOGUES AS PRO-ANGIOGENIC AGENTS

As indicated above, short-term systemic administration of thyroid hormone can induce angiogenesis in an animal model of limb ischemia [43]. We have concluded from the CAM and HDMEC models of angiogenesis that neovascularization induced by iodothyronines is initiated at the integrin receptor for the hormone. Re-formulation of pro-angiogenic thyroid hormone analogues as agents that act exclusively at integrin αvβ3 and that do not enter the cell is desirable and has been achieved as iodothyronine nanoparticulates. It is proposed that these re-formulations may be administered locally for relief of blood vessel stenosis or occlusion by induction of new blood vessels. T_3_, DITPA and GC-1, but not T_4_, are suitable analogues for this use. Local short-term administration may be *via* temporary stents that are coated with thyroid hormone nanoparticles or by catheter proximal to stenoses in large vessels. Temporary stents are proposed because of the adverse events associated with permanent intravascular stents.

The anti-angiogenic property of tetrac, a thyroid hormone antagonist that acts at the integrin receptor for the hormone [13, 45], has been demonstrated in an intact animal model. Nude mouse recipients of a human cancer (medullary carcinoma of the thyroid gland) have been treated with parenteral tetrac or nanoparticulate tetrac for three weeks. The experimental cohort demonstrated a 60% decrease in tumor-associated vascularity, compared to control animals [62].

## CONCLUSIONS

Thyroid hormone analogues are now recognized to be pro-angiogenic. These analogues include T_4_, T_3_, DITPA and GC-1. Studied *in vitro*, the pro-angiogenic activity of these analogues appears to be at a cell surface receptor for the hormone leading to transduction of the hormone signal by MAPK (ERK1/2). The proximate effectors of angiogenesis are bFGF and, very likely, VEGF. That is, an effect of the hormone initiated at the cell surface and that does not necessarily involve access of hormone to the cell interior, culminates in gene expression relevant to neovascularization. In intact animals, hypothyroidism has been shown to result in decreased blood vessel density in brain and heart and this is corrected by thyroid hormone administration. In studies of a hind limb ischemia model in intact rabbit, thyroid hormone administration induces new blood vessel formation. Thus, evidence from intact animals suggests that circulating thyroid hormone supports angiogenesis. It may be useful to test local, short-term administration of thyroid hormone analogues, for example, *via* temporary thyroid hormone-coated stents in management of ischemic tissues. T_4_ should not be used for pro-angiogenic intent in vessels because it causes platelet agglutination, whereas T_3_, DITPA and GC-1 do not have this platelet effect.
